# Gastric ulcer patients are more susceptible to developing gastric cancer compared with concomitant gastric and duodenal ulcer patients

**DOI:** 10.3892/ol.2014.2583

**Published:** 2014-10-02

**Authors:** JUN-BO HONG, WEI ZUO, AN-JIANG WANG, SHAN XU, LU-XIA TU, YOU-XIANG CHEN, XUAN ZHU, NONG-HUA LU

**Affiliations:** 1 Department of Gastroenterology, The First Affiliated Hospital of Nanchang University, Nanchang, Jiangxi 330006, P.R. China; 2 Department of Respiratory Medicine, The First Affiliated Hospital of Nanchang University, Nanchang, Jiangxi 330006, P.R. China; 3 Department of Pathology, The First Affiliated Hospital of Nanchang University, Nanchang, Jiangxi 330006, P.R. China

**Keywords:** concomitant gastric and duodenal ulcers, gastric ulcer, intestinal metaplasia, dysplasia, gastric cancer

## Abstract

Intestinal metaplasia (IM) and dysplasia are precancerous lesions of gastric cancer (GC); however, the prevalence of IM and dysplasia in patients exhibiting single gastric ulcer (GU) and concomitant gastric and duodenal ulcer (CGDU) varies. In the present study consecutive patients who had undergone esophagogastroduodenal endoscopy were retrospectively screened, and those presenting with GU or CGDU were further evaluated for IM and dysplasia. Patients diagnosed with GC or lymphoma and patients with a history of anti-*Helicobacter pylori*, non-steroidal anti-inflammatory medicine (NSAIM), H_2_-receptor antagonist or proton pump inhibitor therapy, were excluded from the present study. Of the 204,073 consecutively screened cases, 8,855 (4.3%) and 2,397 (1.2%) were diagnosed with GU and CGDU, respectively. A total of 1,722 GU and 233 CGDU patients were excluded; thus, 7,133 and 2,164 cases of GU and CGDU, respectively (n=9,297), were included in the present study. IM and dysplasia were observed in 1,348 (14.5%) and 210 (2.3%) patients, respectively. IM was more frequently identified in GU patients compared with CGDU patients (16.4 vs. 8.3%; odds ratio [OR], 2.158; 95% confidence interval [CI], 1.830–2.545; χ^2^=86.932; P<0.001); furthermore, GU patients exhibited significantly more frequent IM compared with CGDU patients at the gastric antrum (14.2 vs. 5.5%; OR, 2.818; 95% CI, 2.199–3.610; χ^2^=72.299; P<0.001), gastric incisura (24.0 vs. 14.1%; OR, 1.922; 95% CI, 1.502–2.432; χ^2^=30.402; P<0.001) and gastric corpus (12.6 vs. 3.3%; OR, 4.259; 95% CI, 1.030–17.609; χ^2^=4.736; P=0.026). Dysplasia was significantly more frequently identified in GU patients compared with CGDU patients (2.7 vs. 0.7%; OR, 4.027; 95% CI, 2.376–6.823; χ^2^=31.315; P<0.001), with GU patients exhibiting significantly more severe dysplasia at the gastric antrum (2.4 vs. 0.7%; OR, 3.339; 95% CI, 1.735–6.425; χ^2^=14.652; P<0.001) and the gastric incisura (2.9 vs. 0.7%; OR, 4.255; 95% CI, 1.694–10.689; χ^2^=11.229; P<0.001). Additionally, mild IM was more frequently identified in GU patients compared with CGDU patients (15.2 vs. 7.1%; OR, 2.353; 95% CI, 1.972–2.807; χ^2^=94.798; P<0.001) and dysplasia of a mild (1.7 vs. 0.6%; OR, 2.807; 95% CI, 1.580–4.987; χ^2^=13.519; P<0.001) or moderate/severe grade (1.1 vs. 0.09%; OR, 11.642; 95% CI, 2.857–47.439; χ^2^=18.896; P<0.001) was more frequent in GU patients compared with CGDU patients. IM and dysplasia were more frequently observed in GU compared with CGDU patients in the present study, which may be associated with an increased probability of developing GC.

## Introduction

Gastric cancer (GC) is one of the leading causes of cancer-related mortality globally, and is the second and fourth most commonly diagnosed cancer in males and females in China, respectively (1,2). Intestinal-type GC develops via a well-defined cascade of precursors: Inflammation, atrophy, intestinal metaplasia (IM), dysplasia and, finally, carcinoma (3). It has been demonstrated that within five years of diagnosis of these precursors, the annual incidence of GC is 0.1, 0.25, 0.6, and 6.0% in atrophic gastritis, IM, mild-to-moderate dysplasia and severe dysplasia patients, respectively (4). Patients exhibiting IM are considered to be at a significantly elevated risk of developing GC (5,6); GC was identified to develop >10.9 times more frequently in the presence of IM compared with in the absence of IM (7). In addition, severe dysplasia patients exhibit a propensity to coexist with or progress to adenocarcinoma (8). These findings indicate that IM and dysplasia are precancerous lesions of GC (9), and may be important markers of the development of GC.

Gastric ulcer (GU) and duodenal ulcer (DU) are types of peptic ulcer disease (PUD). Previous studies have indicated that GU is a precancerous condition of GC (10), that a history of GU confers an increased risk of GC (11), that IM is commonly identified in GU patients (12), and that low- and high-grade dysplasia may develop into adenocarcinoma (13), thus, cases of IM and dysplasia in the presence of GU should be investigated. Furthermore, the risk of non-cardia GC decreased by half in DU patients that had not undergone surgery when compared with DU patients who had received surgery, whilst the risk of non-cardia GC doubled in GU patients that did not undergo surgery compared with GU patients who had received surgery (14). The long-term risk of GC development among patients exhibiting DU was identified to be significantly lower than in patients exhibiting GU (15). Furthermore, an inverse association was identified between the rate of GC and DU incidence (16,17). These findings indicate that DU may be an important protective factor against GC; however, to date, the underlying mechanisms remain unclear. Certain studies have revealed that DU is associated with a reduced risk of histological progression in IM and have identified DU as an independent protective factor against IM progression (18,19). However, the association between DU and dysplasia has not been well reported.

Concomitant gastric and duodenal ulcer (CGDU) disease is a type of PUD characterized by the coexistence of GU and DU. However, the prevalence of IM and dysplasia in CGDU patients and the differences between the histological alterations in GU and CGDU have not yet been elucidated using large cohort studies.

Therefore, the aim of the present study was to determine the underlying mechanisms associated with the incidence of GC in patients with GU and CGDU by demonstrating disparities in the prevalence of IM and dysplasia in GU and CGDU patients.

## Patients and methods

### Inclusion and exclusion criteria

Screening of consecutive patients who underwent esophagogastroduodenal endoscopy due to upper gastrointestinal symptoms at the First Affiliated Hospital of Nanchang University (Nanchang, China) between January 2002 and August 2011 was undertaken. Those who were diagnosed with GU or CGDU were recruited for further analysis. The present study was approved by the Ethics Committee of Nanchang University and informed consent was obtained from each patient.

Endoscopy was used to identify ulcers in the stomach and duodenum; active stage ulcers were identified by a mucosal break (diameter, ≥5 mm) (20) and by evidence of scarring or deformity. GU was diagnosed by the presence of ulcers in the stomach (between the cardia and the pylorus) and CGDU was diagnosed by the presence of ulcers in the stomach and duodenum regardless of complications (for example, gastrointestinal bleeding and perforation). Patients with a history of anti-*Helicobacter pylori* therapy, or treatment with non-steroidal anti-inflammatory medicines (NSAIMs) during the previous three months and H_2_-receptor antagonists or proton pump inhibitors during the previous four weeks, were excluded from the present study. The present study also excluded patients with a histologically determined diagnosis of GC or lymphoma.

Since a number of patients underwent more than one endoscopy during the investigation period, the index endoscopy was defined as the initial endoscopy. However, when a patient exhibited CGDU/GU and/or IM/dysplasia the index endoscopy was defined as the first endoscopy at which CGDU/GU and/or IM/dysplasia was diagnosed. Therefore, each patient was represented by a single index endoscopy (21).

### Histological examinations

During endoscopic examination, biopsies were obtained from ulcer-free regions of the stomach (gastric antrum, n=2; gastric body, n=2; incisura angularis, n=1) and the periphery of each ulcer (n≥2). Histological examinations were performed by pathologists at the First Affiliated Hospital of Nanchang University, and the Sydney System (22) was used to determine histological changes, including chronic and active inflammation, lymphoid aggregates or follicles, atrophy and, particularly, IM, dysplasia and grading (22).

### Statistical analysis

The data are presented as the mean ± standard deviation, a percentage or a rate. The χ^2^ test or Fisher’s exact test with odds ratio (OR) and 95% confidence interval (CI) were used to determine differences in the categorical variables. All statistical analyses were performed using the Statistical Package for the Social Sciences version 17 (SPSS Inc., Chicago, IL, USA). P<0.05 was considered to indicate a statistically significant difference and all reported P-values were two-tailed.

## Results

### Patients

Out of 204,073 consecutive cases, 8,855 (4.3%) and 2,397 (1.2%) were diagnosed with GU and CGDU, respectively. Of the 8,855 GU patients, 1,722 patients were excluded due to GC (n=697) or lymphoma (n=32) diagnosis, a history of anti-*H. pylori* therapy (n=257) and treatment with NSAIMs, H_2_-receptor antagonists or proton pump inhibitors (n=736). In addition, of the 2,397 CGDU patients, 233 were excluded due to GC diagnosis (n=11), a history of anti-*H. pylori* therapy (n=43) and treatment with NSAIMs, H_2_-receptor antagonists or proton pump inhibitors (n=179; Table I). Thus, 9,297 cases (GU, 7133 and CGDU, 2,164; male, 6,775 and female, 2,522; mean age, 45.8±12.1 years) were included in the present study (Table II).

### Prevalence of IM at various sites in GU and CGDU patients

Histological examination diagnosed IM in 1,348 patients (overall prevalence, 14.5%; Table II). The incidence rate (IR) of IM was significantly higher in GU patients compared with in CGDU patients (16.4 vs. 8.3%; OR, 2.158; 95% CI, 1.830–2.545; χ^2^=86.932; P<0.001). Furthermore, compared with CGDU patients, GU patients exhibited a significantly higher frequency of IM at the gastric antrum (14.2 vs. 5.5%; OR, 2.818; 95% CI, 2.199–3.610; χ^2^=72.299; P<0.001), gastric incisura (24.0 vs. 14.1%; OR, 1.922; 95% CI, 1.502–2.432; χ^2^=30.402; P<0.001) and gastric corpus (12.6 vs. 3.3%; OR, 4.259; 95% CI, 1.030–17.609; χ^2^=4.736; P=0.026). However, no difference in the prevalence of IM was identified between GU and CGDU patients at the gastric corpus, fundus or cardia (Table III).

### Prevalence of dysplasia at various sites in GU and CGDU patients

Dysplasia was detected in 210 patients (overall prevalence 2.3%; Table I). The IR of dysplasia was significantly higher in GU patients compared with in CGDU patients (2.7 vs. 0.7%; OR, 4.027; 95% CI, 2.376–6.823; χ^2^=31.315; P<0.001). Furthermore, compared with CGDU patients, GU patients exhibited a significantly higher frequency of dysplasia at the gastric antrum (2.4 vs. 0.7%; OR, 3.339; 95% CI, 1.735–6.425; χ^2^=14.652; P<0.001) and incisura (2.9 vs. 0.7%; OR, 4.255; 95% CI, 1.694–10.689; χ^2^=11.229; P<0.001). However, no difference in the prevalence of dysplasia was identified between GU and CGDU patients at the gastric corpus, fundus or cardia (Table IV).

### Grading of IM and dysplasia in GU and CGDU patients

Mild IM was more frequently detected in GU compared with CGDU patients (15.2 vs. 7.1%; OR, 2.353; 95% CI, 1.972–2.807; χ^2^=94.798; P<0.001); however, no difference was identified in patients with moderate/severe IM (1.2 vs. 1.2%; OR, 0.950; 95% CI, 0.614–1.468; χ^2^=0.054; P=0.822). The frequency of mild (1.7 vs. 0.6%; OR, 2.807; 95% CI, 1.580–4.987; χ^2^=13.519; P<0.001) or moderate/severe (1.1 vs. 0.09%; OR, 11.642; 95% CI, 2.857–47.439; χ^2^=18.896; P<0.001) dysplasia was significantly higher in GU compared with CGDU patients (Table V).

## Discussion

The present study demonstrated that IM and dysplasia were present in 14.5 and 2.3% of patients, respectively. The prevalence of IM and dysplasia in GU patients was significantly higher compared with CGDU patients, indicating that GU patients were more likely to develop GC, whilst the IR of GC may decline in DU patients.

The prevalence of IM and dysplasia in PUD patients has not been well investigated, although it has been identified that in Hong Kong the overall prevalence of IM was 9.4%, with 13.9 and 5.9% of *H. pylori*-positive and *H. pylori*-negative patients, respectively, exhibiting IM (23). Additionally, IM was identified at the highest frequency in GU patients (12). In the present study, the prevalence of IM/dysplasia was 16.4/2.7% and 8.3/0.7% in patients with GU and CGDU, respectively [overall prevalence (all patients), 14.5/2.3%].

A previous study identified that GC developed in 32 (3.3%) GU patients and three (0.68%) DU patients (15) and the frequency with which GC and DU coexisted was 0.1–1.7% (24), indicating that DU is associated to a lesser extent with GC. However, the underlying mechanisms of the low IR of GC in DU patients and the difference in pathological features between GU patients with or without DU remain unclear. Previously, DU has been associated with a reduced risk of histological progression and was identified as an independent protective factor against IM progression (18,19). In the present study, the prevalence of IM was significantly higher in GU patients compared with in CGDU patients, particularly for mild IM. Furthermore, IM was more frequently detected in GU patients at the gastric antrum, incisura and corpus. These findings indicate that GU was more likely to be associated with IM than CGDU, which may contribute to the high incidence of GC in GU patients.

Gastric dysplasia represents the penultimate stage of the gastric carcinogenesis sequence, and is thus a direct neoplastic precancerous lesion (12,25,26). In patients undergoing esophagogastroduodenal endoscopy in the Netherlands, 8% exhibited mild-to-moderate dysplasia and 0.6% exhibited severe dysplasia (4). The risk of gastric tumorigenesis increases with the severity of dysplasia (27) and previous studies have indicated that dysplasia of all grades requires further investigation. However, dysplasia has not been investigated sufficiently in patients with GU and CGDU. Thus, the current study demonstrated that the IR of dysplasia of all grades was significantly higher in GU patients compared with CGDU patients, and that the majority of dysplasia was detected in GU patients at the gastric antrum and incisura. The findings of the present study further demonstrate that dysplasia occurs more frequently in GU patients; thus, GU may increase the risk of gastric carcinogenesis.

A limitation of the present study was that sub-analyses for the subtypes of IM (complete vs. incomplete IM) were not performed, although incomplete IM has previously been proposed as a risk factor for GC development (9). Furthermore, various risk factors of GC (for example, *H. pylori* and diet) were not investigated in the present study and no regular follow-up of the patients occurred, preventing insight into the long-term histological consequences (i.e., dysplasia and GC) of the GU and CGDU patients exhibiting IM/dysplasia. Therefore, the clinical significance of the disparities in historical alterations between GU and CGDU patients requires verification in long-term prospective studies.

In conclusion, IM and dysplasia were present in 14.5 and 2.3% of patients, respectively. IM and dysplasia were significantly more prevalent in GU patients compared with CGDU patients, indicating that GU patients were more prone to developing GC compared with CGDU patients due to the low incidence of IM and dysplasia. Further long-term prospective investigations are required to verify these findings.

## Figures and Tables

**Table I:** Exclusion criteria.

	Patients, n	
	
Criterion	GU	CGDU
Gastric cancer	697	11
Lymphoma	32	0
History of anti-*H. pylori* therapy	257	43
Treatment with NSAIMs, H_2_-receptor antagonists or proton pump inhibitors	736	179

GU, gastric ulcer; CGDU, concomitant gastric and duodenal ulcer; *H. pylori*, *Helicobacter pylori*; NSAIMs, non-steroidal anti-inflammatory medicines.

**Table II:** Patient characteristics.

Variable	Patients, n (%)
Disease	
Gastric ulcer	7133 (76.7)
Concomitant gastric and duodenal ulcer	2164 (23.3)
Gender	
Male	6775 (72.9)
Female	2522 (27.1)
Intestinal metaplasia	1348 (14.5)
Dysplasia	210 (2.3)

Mean age, 45.8±12.1 years; n=9,297.

**Table III:** Prevalence of IM at various gastric sites in GU and CGDU patients.

	GU, n (%)		CGDU, n (%)			
				
Gastric site	IM	No IM	IM	No IM	Odds ratio (95% CI)	P-value
Antrum	563 (14.2)	3410 (85.8)	76 (5.5)	1297 (94.5)	2.818 (2.199–3.610)	<0.001
Incisura	442 (24.0)	1402 (76.0)	102 (14.1)	622 (85.9)	1.922 (1.502–2.432)	<0.001
Corpus	151 (12.6)	1046 (87.4)	2 (3.3)	59 (96.7)	4.259 (1.030–17.609)	0.026
Fundus	4 (6.3)	59 (93.7)	0 (0.0)	5 (100)	0.937 (0.878–0.999)	1.000
Cardia	8 (14.3)	48 (85.7)	0 (0.0)	1 (100)	0.857 (0.770–0.954)	1.000
Total	1168 (16.4)	5965 (83.6)	180 (8.3)	1984 (91.7)	2.158 (1.830–2.545)	<0.001

IM, intestinal metaplasia; GU, gastric ulcer; CGDU, concomitant gastric and duodenal ulcer; n, number of patients; CI, confidence interval. P<0.05 was considered to indicate a statistically significant difference.

**Table IV:** Prevalence of dysplasia at various gastric sites in GU and CGDU patients.

	GU, n (%)		CGDU, n (%)			
				
Gastric site	Dysplasia	No dysplasia	Dysplasia	No dysplasia	Odds ratio (95% CI)	P-value
Antrum	95 (2.4)	3878 (97.6)	10 (0.7)	1363 (99.3)	3.339 (1.735–6.425)	<0.001
Incisura	53 (2.9)	1791 (97.1)	5 (0.7)	719 (99.3)	4.255 (1.694–10.689)	0.001
Corpus	44 (3.7)	1153 (96.3)	0 (0.0)	61 (100)	0.963 (0.953–0.974)	0.268
Fundus	1 (1.6)	62 (98.4)	0 (0.0)	5 (100)	0.984 (0.954–1.105)	1.000
Cardia	2 (3.6)	54 (96.4)	0 (0.0)	1 (100)	0.964 (0.917–1.104)	0.965
Total	195 (2.7)	6938 (97.3)	15 (0.7)	2149 (99.3)	4.027 (2.376–6.823)	<0.001

GU, gastric ulcer; CGDU, concomitant gastric and duodenal ulcer; CI, confidence interval. P<0.05 was considered to indicate a statistically significant difference.

**Table V:** IM and dysplasia grading in GU (n=7,133) and CGDU (n=2,164) patients.

	IM, n (%)				Dysplasia, n (%)			
						
Grade	GU	CGDU	Odds ratio (95% CI)	P-value	GU	CGDU	Odds ratio (95% CI)	P-value
Mild	1083 (15.2)	153 (7.1)	2.353 (1.972–2.807)	<0.001	119 (1.7)	13 (0.6)	2.807 (1.580–4.987)	<0.001
Moderate/severe	85 (1.2)^a^	27 (1.2)^b^	0.950 (0.614–1.468)	0.822	76 (1.1)^c^	2 (0.09)^d^	11.642 (2.857–47.439)	<0.001

Including

a six cases of severe IM;

b three cases of severe IM;

c 47 cases of severe dysplasia; and

d one case of severe dysplasia.

IM, intestinal metaplasia; GU, gastric ulcer; CGDU, concomitant gastric and duodenal ulcer; n, number of patients; CI, confidence interval.
